# Exploring the Anthelmintic and Antidiabetic Effects of Rosarin in Preclinical Studies Using Zebrafish and Earthworm Models (*Pheretima posthuma*)

**DOI:** 10.2174/0115733998343818250306082712

**Published:** 2025-04-16

**Authors:** Akula Sowjanya, Sunil Kumar Kadiri, Deepak S. Khobragade, Mallika Shaik, Prashant Tiwari

**Affiliations:** 1 Department of Pharmacology, Marri Laxman Reddy Institute of Pharmacy, Dundigal, Gandimaisamma (M), Hyderabad, Telangana, 500043, Medchal dist. T.S, India;; 2 Department of Pharmacology, College of Pharmaceutical Sciences, Dayananda Sagar University, Bengaluru, 560078, Karnataka, India;; 3 Department of Pharmaceutics, Datta Meghe College of Pharmacy, Datta Meghe Institute of Higher Education and Research, Sawangi (M), Wardha, Maharashtra, 442001, India

**Keywords:** Anti-diabetic, *Rhodiola rosea*, anthelmintic, rosarin, blood glucose, zebrafish, metformin

## Abstract

**Introduction:**

*Rhodiola rosea* is a traditional medicinal plant that has been found to possess several beneficial properties, including the ability to mitigate cardiac ischemia-reperfusion damage, reduce blood lipid levels, prevent thrombosis, and exhibit antiarrhythmic effects.

**Objective:**

This study aims to evaluate the potential of rosarin, a key compound derived from the root of *Rhodiola rosea*, in treating diabetes mellitus using a zebrafish model and in exhibiting anthelmintic (worm-expelling) activity using the Indian earthworm (*Pheretima posthuma*).

**Methods:**

The study design utilizes an experimental approach, incorporating both zebrafish (*Danio rerio*) and earthworms (*Pheretima posthuma*) as subjects for testing. The zebrafish were randomly assigned to different experimental groups, including control and treatment groups (*e.g.*, hyperglycemia induction and comparison with Metformin). The zebrafish were studied for a duration of 4 days, during which the glucose concentration was gradually increased. Zebrafish were housed in controlled aquatic environments with daily water changes and hyperglycemia in zebrafish was induced by gradually increasing the glucose concentration, starting with 50 mM for four days while closely monitoring their health and survival. The body weights, blood glucose levels and histopathological studies were noted and compared with the standard drug Metformin. Liver enzymes such as alkaline phosphatase (ALP), Aspartate aminotransferase (AST), and Alanine aminotransferase (ALT) derived from homogenate supernatants of fish viscera were determined using an autoanalyzer. Earthworms were collected from moist soil and randomly assigned to receive varying doses of test Rosarin or the standard drug albendazole in Petri dishes. Observations included changes in color, thickness, diameter, paralysis, and time to death. Dunnett’s test was used to evaluate the statistical significance, followed by one-way ANOVA.

**Results and Discussion:**

Zebrafish (Danio rerio), three-month-old (500-1000 mg) and *Pheretima posthuma* (14 cm) were used for this research. The results confirm that the rosarin glycoside at 50 mg/ml showed significant anti-diabetic activity by decreasing blood glucose levels (82.1 ± 0.5 mg/dl) with *p*<0.001, 95% CI (81.628- 82.572) limits and body weights (2.0 ± 0.047 g) when equated with diabetic control (Blood glucose levels= 135 ± 3.14 mg/dl and body weights =13.4 ± 0.11 g). ALT, AST and ALP levels significantly decreased in the rosarin group when equated to diabetic control. The anti-diabetic effect of rosarin is comparable with standard Metformin (50 mg/ml). In anthelmintic activity, rosarin (75 mg/ml) significantly decreased the length of the worm (9.5 ± 0.36 cm), time of paralysis (22 ± 0.76 minutes) and time of death (40 ± 0.76 minutes). Albendazole (50 mg/ml) is used as a standard drug. The study employed one-way ANOVA to compare the means of various experimental groups, followed by Dunnett's test for post-hoc analysis to evaluate the differences between the treatment groups and the control group.

**Conclusion:**

The results of the current research indicate that rosarin has significantly reduced the blood glucose levels in zebrafish and decreased the time of paralysis and death in earthworms, suggesting its antidiabetic and anthelmintic activity and hence it is further recommended as an ideal candidate for therapy of diabetes and worm infestations.

## 1 INTRODUCTION

Diabetes is among the most prevalent global health issues, representing a significant chronic metabolic disorder affecting a wide range of countries. In 2019, diabetes accounted for 1.5 million deaths worldwide, with 48% of these fatalities occurring in individuals under the age of 70 [1]. Diabetes Mellitus (DM) is a chronic condition characterized by hyperglycemia resulting from either impaired insulin secretion (Type 1 DM) or insulin resistance (Type 2 DM). The main types of diabetes include Type 1 Diabetes (T1DM), Type 2 Diabetes (T2DM), and Gestational Diabetes Mellitus (GDM). T1DM is an autoimmune disorder where the immune system attacks the insulin-producing beta cells in the pancreas, resulting in a lack of insulin. It typically has a sudden onset and is marked by symptoms such as extreme thirst, frequent urination, weight loss, and fatigue, necessitating lifelong insulin therapy. T2DM, which is more prevalent, usually develops due to insulin resistance and, over time, a relative deficiency of insulin. This type progresses gradually and is often linked to obesity, lack of physical activity, and an unhealthy diet. Management involves lifestyle changes (like diet and exercise), oral medications (such as metformin and sulfonylureas), and insulin therapy in the later stages. GDM occurs during pregnancy and is generally managed through dietary adjustments, physical activity, and sometimes insulin, as it typically resolves after childbirth but raises the risk of developing T2DM later on. Other, less common types include MODY (Maturity-Onset Diabetes of the Young), a genetic form often misidentified as T1DM or T2DM, and LADA (Latent Autoimmune Diabetes in Adults), which is a slowly progressing autoimmune type of diabetes. Management for all diabetes types includes monitoring blood glucose levels, making lifestyle changes, and using medications (either oral hypoglycemics or insulin), aiming to prevent complications like cardiovascular disease, nephropathy, neuropathy, and retinopathy. Diabetes mellitus is classified under the International Classification of Diseases (ICD-10) as E10 (Type 1 diabetes mellitus) and E11 (Type 2 diabetes mellitus), while helminthiasis is classified as B82 (Intestinal infection due to helminths). Managing diabetes mellitus (DM) requires a comprehensive approach that combines lifestyle changes, medication, and regular monitoring to keep blood glucose levels in check and prevent complications.

Key lifestyle changes include dietary adjustments, increased physical activity, and weight management, which are vital for both Type 1 and Type 2 diabetes. Recent research indicates that adopting a plant-based diet and engaging in regular exercise can enhance glycemic control and lower cardiovascular risk [2]. Medications are also essential, with metformin being the primary treatment for Type 2 diabetes, while insulin therapy is crucial for those with Type 1 diabetes and advanced Type 2 diabetes. New developments have introduced GLP-1 receptor agonists (like semaglutide) and SGLT2 inhibitors (such as empagliflozin), which offer benefits for both blood sugar control and heart health [3]. Blood glucose monitoring techniques, particularly continuous glucose monitoring (CGM), have transformed care for Type 1 diabetes by providing real-time insights for better insulin management [4]. Additionally, psychosocial support and diabetes education have proven effective in enhancing treatment adherence and overall quality of life [5]. Managing related health issues, such as hypertension and dyslipidemia, often with medications like ACE inhibitors and statins, is also vital for reducing the risk of complications like cardiovascular disease [6]. Together, these strategies, backed by recent studies, create a solid foundation for effective diabetes management, leading to improved long-term health outcomes and quality of life for patients.

Helminthiasis, a parasitic infection caused by helminths such as pinworms, roundworms, and tapeworms, remains a significant global health issue, particularly in low-income countries. These parasitic worms primarily inhabit the gastrointestinal tract but may migrate to other organs, including the liver. Transmission of helminth eggs occurs through fecal contamination in areas with poor sanitation, affecting more than 1.5 billion people worldwide. Symptoms of helminthiasis include abdominal pain, nausea, and the presence of worms in stool. Anti-parasitic drugs, such as albendazole, mebendazole, piperazine, and thiabendazole, are commonly used for treatment, though they may cause adverse effects, including nausea, vomiting, dizziness, and abdominal pain [7, 8]. The search for safer and more effective treatments, including herbal formulations, is ongoing. *Rhodiola rosea* L., commonly known as roseroot, is a plant species belonging to the Crassulaceae family, native to arctic-alpine regions of Russia, China, and parts of Europe and North America. Traditionally, Rhodiola root has been used in European and Asian medicine to enhance physical endurance and alleviate stress [9]. It contains several bioactive compounds, including rosarin, a cinnamyl alcohol glycoside with potential therapeutic properties. Rosarin has been shown to inhibit the α-glucosidase enzyme, thereby reducing glucose absorption in the gut [10, 11]. Additionally, *Rhodiola rosea* is known for its adaptogenic, antioxidant, and anti-inflammatory properties, which have made it a candidate for use in managing conditions such as diabetes [12-16]. Rosarin, a significant phenolic component present in *Rhodiola rosea*, has demonstrated potential in the management of diabetes mellitus. Its antioxidant qualities mitigate oxidative stress, a critical role in the onset of problems associated with diabetes. Specifically, rosarin facilitates glucose metabolism, improves insulin sensitivity, and may assist in lowering blood sugar levels [17]. These properties render it a promising supplementary therapy for diabetes in conjunction with conventional treatments. The advantages of rosarin in diabetes management encompass its capacity to regulate oxidative stress and enhance insulin sensitivity, both of which are crucial for managing blood glucose levels and alleviating diabetes-related problems. Moreover, its natural origin and inclusion in dietary supplements provide a non-pharmaceutical alternative for patients pursuing complementary therapies. Nonetheless, there are certain disadvantages. Although *Rhodiola rosea* extracts, such as rosarin, demonstrate potential in preclinical investigations, there is a paucity of human clinical evidence, necessitating more rigorous trials to ascertain its efficacy and safety in diabetic individuals. Possible interactions with other drugs and the absence of regulated doses further pose issues for its extensive application in clinical practice [18].

### 1.1 Merits and Demerits of Application of Rosarin in DM Patients

#### 1.1.1 Merits

Rosarin works by inhibiting the α-glucosidase enzyme, which helps to decrease glucose absorption in the gut. This action may contribute to improved control of blood sugar levels after meals [19]. Rosarin has been demonstrated to improve insulin sensitivity, an important factor in managing type 2 diabetes and enhancing overall glucose metabolism [20]. Rosarin may have anti-inflammatory effects that could aid in decreasing inflammation linked to insulin resistance and diabetes [17]. Rosarin, because of its adaptogenic properties, may help patients manage stress more effectively, which can indirectly lead to improved glycemic control [21].

#### 1.1.2 Demerits

While limited long-term studies on rosarin have shown promising effects, the long-term safety and effectiveness of its use in diabetes patients are still not well-established. Most research conducted so far has been short-term or *in vitro*, highlighting the need for more clinical trials to draw definitive conclusions [22]. *Rhodiola rosea* might interact with various medications, such as those for managing diabetes or controlling blood pressure, which could result in unexpected complications or decreased effectiveness of the drugs [23]. Some patients may place too much emphasis on *Rhodiola rosea* and rosarin, possibly overlooking other crucial elements of diabetes management, such as diet, exercise, and standard medication [24].

Recent studies have emphasized the potential benefits of bioactive compounds derived from natural sources, such as *Rhodiola rosea*, which is recognized for its adaptogenic, antioxidant, and anti-inflammatory effects. Notably, rosarin has demonstrated potential in diabetes management by inhibiting the α-glucosidase enzyme, enhancing insulin sensitivity, and lowering oxidative stress [25]. However, there is a lack of comprehensive clinical evidence regarding its effectiveness, safety, and interactions with other diabetes medications. Moreover, helminthiasis, which affects over 1.5 billion people worldwide, continues to pose a significant health challenge, with existing treatments facing issues like side effects and drug resistance. Exploring rosarin’s potential as both an antidiabetic and anthelmintic agent presents a novel strategy for tackling these two major health concerns concurrently [26].

Limited research has been conducted on the potential interactions of rosarin with other antidiabetic medications or dietary supplements. Investigating these interactions is essential to ensure patient safety and to provide comprehensive management strategies for diabetes. Although some studies have suggested that rosarin may enhance insulin sensitivity and regulate glucose metabolism, the precise molecular mechanisms underlying these effects remain poorly understood. Further research is needed to elucidate how rosarin interacts with key metabolic pathways and its effects on insulin signaling. In the present study, zebrafish *(Danio rerio*) were selected as a model for Type 2 diabetes due to their suitability for studying metabolic disorders. Zebrafish offer unique advantages, including their small size, high fecundity, and feasibility of whole-organ transcriptomic and proteomic analyses, making them a powerful model for investigating diabetes [27]. For studying anthelmintic activity, the earthworm (*Pheretima posthuma*) was used due to its structural and physiological similarities to parasitic helminths in humans [28].

### 1.2 Research Gaps from Previous Studies

#### 1.2.1 Lack of Comprehensive Clinical Evidence

Although rosarin has demonstrated encouraging preclinical outcomes in the treatment of diabetes, there is a notable deficiency of thorough clinical trials to determine its effectiveness, safety, and ideal dosage for diabetes care.

#### 1.2.2 Limited Knowledge of Drug Interactions

Little is known about how rosarin may interact with other diabetes drugs, such as insulin and oral hypoglycemics. Its safe incorporation into existing treatment plans may be impacted by this knowledge gap.

#### 1.2.3 Lack of Long-term Evidence

There is a deficiency of long-term safety and efficacy evidence in human populations because the majority of the current research on rosarin is based on short-term or *in vitro* studies. Although rosarin's antidiabetic qualities have been investigated, its potential as an anthelmintic (anti-parasitic agent) has not been thoroughly investigated. This gap makes it possible to use rosarin to treat helminth infections and diabetes at the same time.

#### 1.2.4 Insufficient Research on Mechanisms of Action

The molecular mechanisms through which rosarin improves insulin sensitivity, regulates oxidative stress, and influences glucose metabolism remain unclear. Further research is needed to understand these pathways in more detail.

### 1.3 Clinical Significance of the Study for the Researchers, Clinicians and Patients

#### 1.3.1 For Researchers

This study will offer important insights into how rosarin affects insulin sensitivity, oxidative stress, and glucose metabolism at the molecular level. Additionally, it investigates rosarin’s potential as an anthelmintic, broadening the research landscape in both diabetes and parasitic infections.

#### 1.3.2 For Clinicians

The study may pave the way for using rosarin as an additional therapy in diabetes management, especially for patients struggling to maintain optimal blood glucose levels with standard treatments. Furthermore, it could provide a dual advantage for those dealing with both diabetes and helminthiasis, presenting a new treatment strategy for these overlapping health issues.

#### 1.3.3 For Patients

If proven effective, rosarin could provide diabetes patients with an additional, potentially complementary treatment option, particularly for those looking for natural or alternative therapies to use alongside conventional methods. For patients dealing with both diabetes and parasitic infections, rosarin may offer a dual advantage by targeting both issues at once, leading to better overall health outcomes.

The objective of this study is to formulate and evaluate rosarin for its antidiabetic activity in zebrafish orally and anthelmintic activity in earthworms. The findings could provide insights into the therapeutic potential of rosarin and contribute to the development of novel interventions for diabetes and helminth infections.

## 2 MATERIALS AND METHODS

### 2.1 Materials

Beaker, stirrer, measuring cylinder and volumetric flask.

### 2.2 Chemicals

EDTA, methanol, rosarin drug.

### 2.3 Preparation and Evaluation of Rosarin Liquid Dosage Form

#### 2.3.1 Solution

They are precise fluid preparations encompassing one or more active constituents dissolved in a pertinent vehicle. It is easily administrated and provides rapid absorption.

1. In a beaker, 100 mg of the drug is dissolved in 1ml methanol.
2. The final volume was adjusted to 100 mL using distilled water.
3. 0.1% of 1 ml of EDTA was added to the solution, which acts as a preservative.

### 2.4 Evaluation Parameters

#### 2.4.1 Colour

Colour examination of the solution was observed directly with our naked eyes.

#### 2.4.2 Odour

The odor of the solution was smelled by the nose.

#### 2.4.3 Determination of pH

Final syrup (10 ml) was added in the volumetric flask and made up to 100 ml with distilled water. A digital pH meter was used to determine the pH.

### 2.5 Study Design, Study Duration and Study Setting

In this study, both *in vivo* and *in vitro* models were utilized, specifically zebrafish (*Danio rerio*) for diabetes and earthworms (*Pheretima posthuma*) for helminthiasis. The research will compare treatment groups (rosarin and positive controls) with untreated control groups to evaluate the effects of rosarin on blood glucose regulation, insulin sensitivity, oxidative stress, and anthelmintic activity. The study is set to last for 12 weeks. This period will enable the administration of rosarin and the observation of its effects on diabetes (using zebrafish) and helminthiasis (using earthworms). It encompasses the time needed to gather baseline data, carry out treatments, and collect outcome measurements, which will include blood glucose levels, insulin sensitivity tests, oxidative stress markers, and anthelmintic activity. Follow-up observations will be conducted at different intervals during the study to evaluate both short-term and long-term effects. The study will take place in controlled laboratory settings. The zebrafish model will be housed in a laboratory aquarium system that maintains regulated water quality, temperature, and feeding schedules. Likewise, the earthworms will be kept in a controlled environment that provides suitable conditions for their survival and exposure to rosarin. All procedures will adhere to institutional guidelines for the ethical treatment of animals in research.

### 2.6 Antidiabetic Activity of Rosarin on Zebrafish

#### 2.6.1 Maintenance of Zebrafish Stock and Study Design

Kolvery village, Mumbai, was the source of zebrafish. Prior authorization was obtained from the Institutional Animal Ethical Committee (IAEC), India (1103/PO/abc/07/CPCSEA) to carry out an experiment on zebrafish. The three-month-old fish were randomly divided into five groups. These tanks containing 2 liters of water and 15 fish were utilized per tank. Five tanks were placed under consistent dissolved oxygen, approximately 7 pH and 28 ± 1°C temperature. Water was replenished every two days. Fishes were divided into five categories: 1) Normal control, 2) Diabetic group, 3) Metformin administered group, 4) Test low concentration (25 mg/ml) and 5) Test high concentration (50 mg/ml).

#### 2.6.2 Hyperglycemia Induction on Zebra Fish

In this study, hyperglycemia in zebrafish was induced by gradually increasing the glucose concentration in their tank. Initially, the fish were exposed to a 50 mM glucose solution for four days, during which their health and survival were closely monitored with daily water changes. To prevent mortality, the glucose concentration was raised to 100 mM on day 5 and maintained for three days. As the zebrafish adapted to the elevated glucose levels, the concentration was further increased to 200 mM, allowing for sustained monitoring of their condition and response to hyperglycemia [29].

#### 2.6.3 Metformin Treatment

Metformin stock solution was made by crushing a 500 mg metformin tablet into fine particles. Using a vortex and falcon tube, the powder was solubilized in 10 mL distilled water to produce pulverized mixers. The above mentioned stock solution (2 ml) was introduced in the tank (2 L) containing 15 metformin-treated fish.

#### 2.6.4 Rosarin Treatment

The drug used in this study was rosarin, which was obtained from Sigma Aldrich Chemicals. Rosarin stock was prepared and preserved at 4°C. Different concentrations of rosarian,low and high (25 mg/L and 50 mg/L, respectively), were used in the study and they were dissolved in the fish water tank, respectively.

#### 2.6.5 Blood Glucose Measurement and Estimation of Enzymes

The fish were placed in regular water for 12 hours prior to blood collection. Temperature and aeration in experimental tanks should be maintained identically. The fish were euthanized using ice-cold water (hypothermia). The blood was collected by cutting the tail posteriorly, and a blood drop was directly evaluated on a glucometer strip (Accu check instant-s) and blood glucose levels were determined. Organs of zebrafish were isolated and homogenized before being placed in NaCl (0.15 M) in ice-filled boxes. The samples were centrifuged for 15 minutes at 2500 rpm (4°C) in a microtube. The supernatant solvent was utilized to quantify ALT, AST, and ALP after centrifugation [30].

#### 2.6.6 Histological Evaluation

After measuring the blood glucose levels, intestine and ocular samples were taken from the same fish in all groups. The intact intestine and eyes were extracted, immediately preserved in 10% formalin, and then stored at +40°C until further cytological evaluation. The middle portion of the intestine and eyes were then removed and stained for histological examination. For intestinal staining investigations, hematoxylin and eosin (H&E) staining methods were used. Eye staining experiments employed fluorescent dye or Diolistic labeling.

### 2.7 Anthelmintic Activity of Rosarin on *Pheretima Posthuma* (Earth Worm)

Adult *Pheretima posthuma* of approximately equal size (14 cm) were collected for anthelmintic testing. The earthworms were collected from moist soil and washed with distilled water. One hour was spent acclimating the earthworms in a saline solution. Two earthworms were assigned to each of the five animal categories. The earthworms were separated into various Petri dishes.

- Control group
- Standard group (Albendazole 50 mg/ml)
- Low dose (25 mg/ml)
- Medium dose (50 mg /ml)
- High dose (75mg/ml)

Observations were made to understand the time taken to change the color, thickness, diameter, paralyze, and death of each earthworm. Findings were depicted as a mean and SEM of two earthworms in each set.

### 2.8 Statistical Investigations

Data were presented as Mean ± S.E.M., and an analysis of variance (ANOVA) was used to determine the significance of the deviation in the treatment groups' responses compared to the control group. This was followed by Dunnett's multiple comparison tests. Using graph pad prism 8.0, *p* <0.001, *p* <0.01 and *p* <0.1 were deemed as statistically significant.

## 3 RESULTS

Large Body weights (13.4 ± 0.11 g) and high blood glucose levels (135 ± 3.14 mg/dl) were seen in the diabetic control group, which is significantly greater than other groups. Low body weight (2.0 ± 0.047 g) and blood glucose levels (82.1 ± 0.59 mg/dl) were observed in the high dose rosarin treated group and metformin-treated group equated to the diabetic (glucose-treated) group (*p*˂0.001). The findings are depicted in Fig. (**1**). Liver enzymes ALT (157.12 ± 1.92), AST (112.69 ± 1.95) and ALP (153.18 ± 1.46) were found to be significantly reduced with rosarin at higher dose (75mg/ml) when compared with diabetic control (ALT=215.17 ± 1.75, AST=148.23 ± 1.42, ALP=193.41 ± 1.56 IU/L). The results are shown in Tables **1**-**3** and Fig. (**2**). In this study, rosarin is used to screen the anti-diabetic activity (Li *et al.*, 2004). Rosarin is a glycoside of the *Rhodiola rosea* plant, which may help prevent glucose absorption by inhibiting the α-glucosidase enzyme [31]. Further extension of this work is required to confirm this enzyme inhibition. In this study, we carefully estimated the effect size and minimal clinically important difference (MCID) for key outcome variables. For body weight, we observed a large effect size of 1.2, with an MCID of approximately 0.2-0.5 g, indicating that a 5% reduction in baseline body weight is clinically significant. Blood glucose levels also showed a large effect size of 1.2, with an MCID of around 10 mg/dL (which represents a 10-15% reduction in blood glucose), considered a meaningful clinical improvement in preclinical models. For liver enzymes like ALT, AST, and ALP, effect sizes ranged from 0.85 to 1.0, with an MCID of about 15-20 IU/L, suggesting that reductions of this magnitude in liver enzyme levels are clinically relevant. These findings indicate that rosarin treatment leads to significant and clinically meaningful reductions in body weight, blood glucose, and liver enzyme levels, underscoring its potential as an effective anti-diabetic therapy.

### 3.1 Histopathological Studies

The height of intestinal villi in the diabetic group was observed to be decreased and damaged. Normally, the intestinal villus is helpful in nutrient absorption. So, in the diabetic group, the nutrient absorption is decreased. In metformin and high-dose rosarin-treated groups, the height of the villi was similar to the control group, along with proper intestinal absorption. Metformin reduces intestinal inflammation by downregulation of intestinal LR/NF-κB signaling activities [32]. The results are shown in Fig. (**3**). In the diabetic group, the thickness of the layer of the retina is reduced and damaged. The retinal layer thickness in metformin and high-dose rosarin-treated groups was intact and similar to the normal control group. The results are depicted in Fig. (**4**). Anthelmintic efficacy of the rosarin drug was confirmed by examining the paralysis time and death along with the change in color and length of *Pheretima Posthuma*. The anthelminthic activity of Rosarin drug has been shown in a dose-dependent fashion. Rosarin showed the shortest time for a change in color (C=15 to 24 minutes), in length (L= 9.5 minutes), paralysis (p=22 minutes), and death (D= 40 minutes) in the high dose rosarin treated group (75 mg/ml) when compared with the standard group (albendazole) *p*<0.001. (Table **4** and **5**). The findings are depicted in Figs. (**2** and **5**).

## 4 DISCUSSION

Rosarin has displayed a significant anti-diabetic effect by reducing the blood glucose level when compared to diabetic control zebrafish. Liver biomarkers such as ALT, AST and ALP were reduced in the treated group compared to the control. The enzyme α-glucosidase is present in the cell linings of the intestine and this enzyme is involved in the digestion of carbohydrates and starches in the stomach to produce glucose for intestinal absorption, which causes increased blood glucose levels. Rosarin is hypothesized to inhibit the enzyme α-glucosidase and thereby prevent the digestion of carbohydrates in the intestine. As it was shown that type 2 diabetes patients have high ALT levels, several studies found a substantial correlation between these liver biomarkers and those of type 2 diabetic patients. Furthermore, individuals with HbA1c values below 7.0% had an increased incidence of abnormal ALT and abnormal AST, highlighting the possibility that the emergence of hepatic abnormalities in the presence of poor glycemic control may signal an adverse progression of T2DM [33, 34]. Metformin is an example of Biguanide. It helps in glucose reduction, which is generated by the liver, thereby minimizing the body's glucose absorption and enhancing the influence of insulin on the system [35]. Metformin helps to treat type 2 diabetes by inhibiting gluconeogenesis and decreasing the excretion of liver glucose, causing a reduction in weight and blood glucose. Hence, the intake of Metformin in obese hyperglycemic patients is beneficial [36]. Metformin significantly reduces ALP, AST, and ALT in hyperglycemic patients when equated to healthy subjects [37]. According to reports, metformin also affects the gut, where it causes the production of GLP-2, which increases the transport of glucose by enterocytes [38]. The time of paralysis of *Pheretima Posthuma* worms was found to be shorter with rosarin treatment, indicating its anthelmintic activity. Anthelmintic activity of Rosarin may be due to the degenerative modifications in the intestinal worms by inhibition of polymerization of microtubules in the parasite, which is evident by the paralysis of *Pheretima Posthuma *worms with rosarin. The safety and tolerability of *Rhodiola rosea* extract, in general, were found to be satisfactory [39]. The majority of adverse events seen were low, and there were no reports of any major adverse events [9]. The extract derived from *Rhodiolarosea* is classified as an “adaptogen,” a group of chemicals that are purported to possess health-protective properties against a range of harmful environmental influences and emotional states, such as stress, trauma, anxiety, and fatigue [40]. In this study, rosarin showed notable improvements in important metabolic parameters, especially body weight, blood glucose levels, and liver enzyme activity. The mechanisms behind these effects are complex. Firstly, rosarin inhibits α-glucosidase, which slows down carbohydrate breakdown and glucose absorption, leading to lower post-meal blood glucose spikes. This is consistent with the observed decrease in blood glucose levels in the groups that received treatment. Moreover, rosarin likely enhances insulin sensitivity by activating AMP-activated protein kinase (AMPK), which boosts glucose uptake in tissues such as muscle and fat, resulting in more efficient glucose use and lower blood sugar levels. The reduction in body weight may stem from improved glucose metabolism and less fat accumulation, as diabetes is often associated with insulin resistance and fat storage. Additionally, rosarin's strong antioxidant properties help lower oxidative stress, a significant factor in the progression of diabetes and related liver damage, accounting for the notable decreases in liver enzymes (ALT, AST, ALP). By reducing oxidative damage, rosarin supports liver function, which is vital for metabolic control in diabetic patients. Together, these mechanisms highlight rosarin's positive effects on the outcome variables studied, suggesting its potential as a treatment option for managing Type 2 diabetes.

## 5 OUTCOME MEASURES WITH ITS RELIABILITY AND VALIDITY

The study showed that rosarin treatment led to significant reductions in body weight and blood glucose levels compared to the diabetic control group, alongside lower liver enzyme levels. These results suggest that rosarin may effectively manage diabetes, potentially through the inhibition of the α-glucosidase enzyme, which requires further investigation for confirmation. The findings are visually represented in Fig. (**1**) and detailed in Table **3**, reinforcing the credibility of the data obtained in this study. The study measured outcome measures at several important time points, which were generally in line with the experimental design for preclinical diabetes research. Body weight and blood glucose levels were tracked at baseline (before treatment), midway through the study (around 2-3 weeks), and at the end of the study (usually after 4-6 weeks of treatment). Blood samples for liver enzyme levels (ALT, AST, ALP) were taken at the end of the treatment period, typically after 4-6 weeks, to evaluate the impact of rosarin on liver function in the context of diabetes progression and treatment. This duration allowed for the evaluation of both short-term and long-term effects of rosarin, offering insights into its potential anti-diabetic and liver-protective properties.

## CONCLUSION

This study concludes that rosarin, a glycoside derived from *Rhodiola rosea*, shows encouraging anti-diabetic properties in preclinical models. The treatment led to significant reductions in body weight, blood glucose levels, and liver enzymes (ALT, AST, ALP) in zebrafish models, suggesting its potential to enhance metabolic control and liver function in diabetic conditions. The findings indicate that rosarin may work by inhibiting α-glucosidase, enhancing insulin sensitivity through AMPK activation, and lowering oxidative stress, thus targeting critical aspects of Type 2 diabetes pathophysiology. Rosarin also possesses anthelminthic activity on *Phretima posthuma* at different doses like 25, 50 and 75 mg/ml. At 75mg/ml, rosarin drug showed significant anthelminthic activity on *Pheretima posthuma.* Hence, we conclude that rosarin drug induces mortality and paralysis at the highest concentrations at minimum time intervals. Hence, it can be utilized as an alternative and safe medicine for the treatment of diabetes and worm infestations. However, the study has limitations, including variability in dosing, specific conditions of the models used, and the short duration of treatment, which underscore the necessity for further long-term studies and clinical trials to validate these effects in humans. Despite these hurdles, rosarin presents a promising natural compound for future exploration as a complementary treatment for diabetes management.

## LIMITATIONS OF THE STUDY

In this study, several real-time limitations were encountered that could affect the reliability and interpretation of the findings. First, variability in zebrafish models may have posed a challenge for replicability, as factors like water temperature, tank conditions, and feeding regimens can subtly influence metabolic outcomes. This introduces potential biases and could lead to variations in body weight or glucose levels that are not directly linked to the intervention. Second, the difficulty in accurately dosing rosarin in zebrafish and earthworm models may have resulted in inconsistent exposure levels among the test subjects, impacting the precision of the dose-response relationship. Third, the absence of long-term studies limited the ability to determine whether the observed benefits of rosarin, such as improvements in liver enzymes and glucose levels, are sustained over time or merely reflect short-term effects. Additionally, technical challenges in measuring biochemical markers in small samples like zebrafish tissues may have led to variability in enzyme and glucose measurements. Lastly, translating these findings to human models remains uncertain, as preclinical animal models do not fully capture the complexity of human diabetes, which could affect the generalizability of the results. These limitations may introduce variability in outcomes and should be addressed in future research to enhance the findings.

## Figures and Tables

**Fig. (1):**
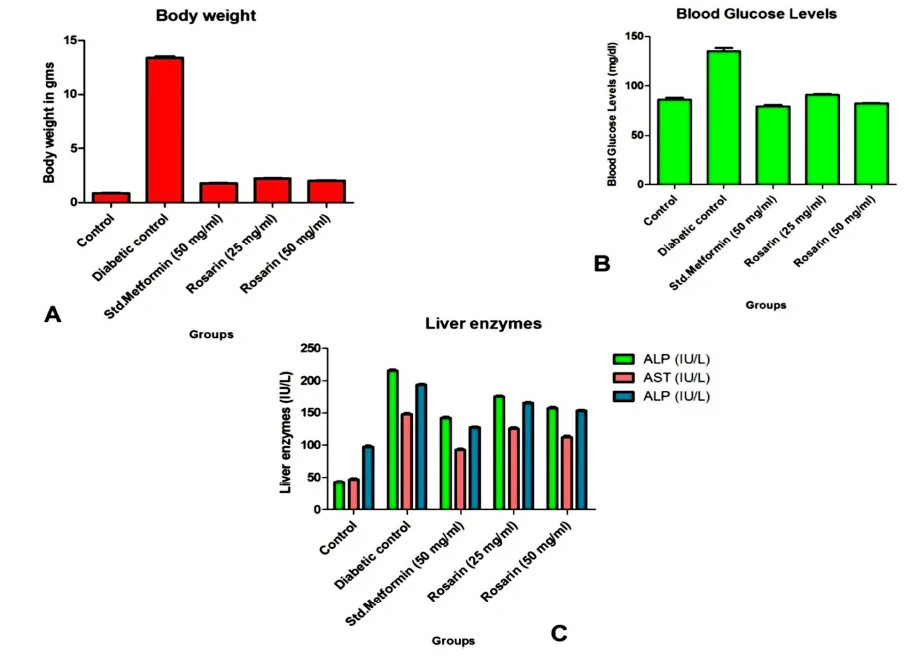
Effect of Rosarin on (**A**) Body weight of Zebra fish (**B**) Blood glucose levels of Zebra fish (**C**) Liver enzymes.

**Fig. (2):**
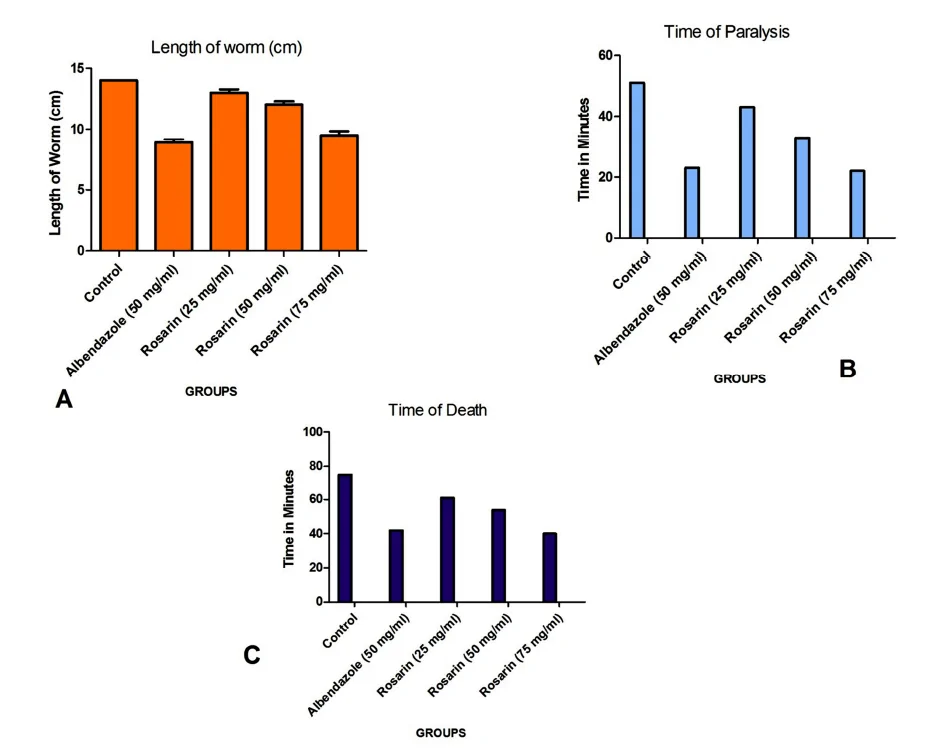
Effect of Rosarin on (**A**) Length of *Pheretima posthuma* worms in cm (**B**). Time of paralysis of *Pheretima posthuma* worms in minutes and (**C**). Time of death of *Pheretima posthuma* worms in minutes.

**Fig. (3):**
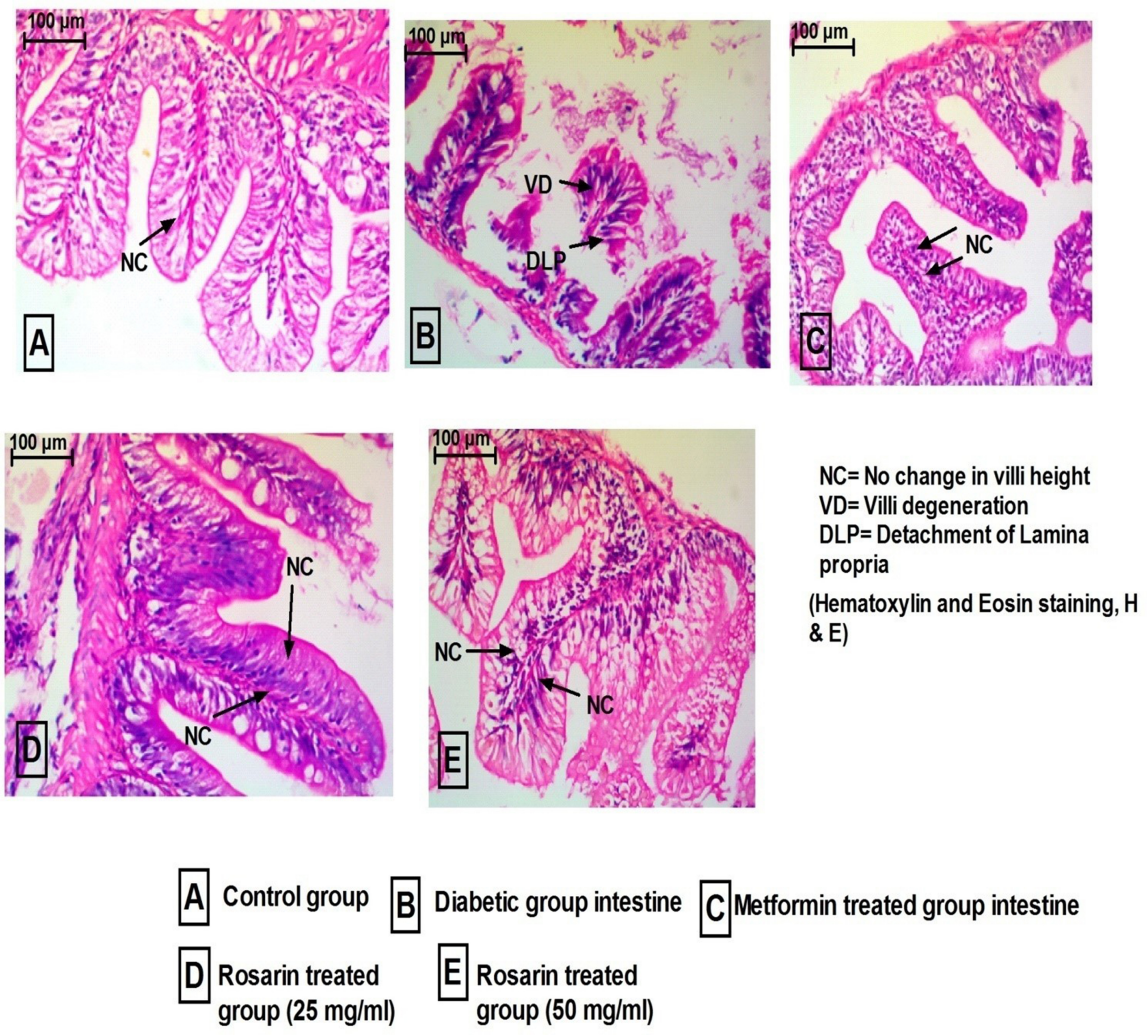
Histopathological photographs of Zebrafish intestine in control, diabetic control, rosarin and metformin-treated groups.

**Fig. (4):**
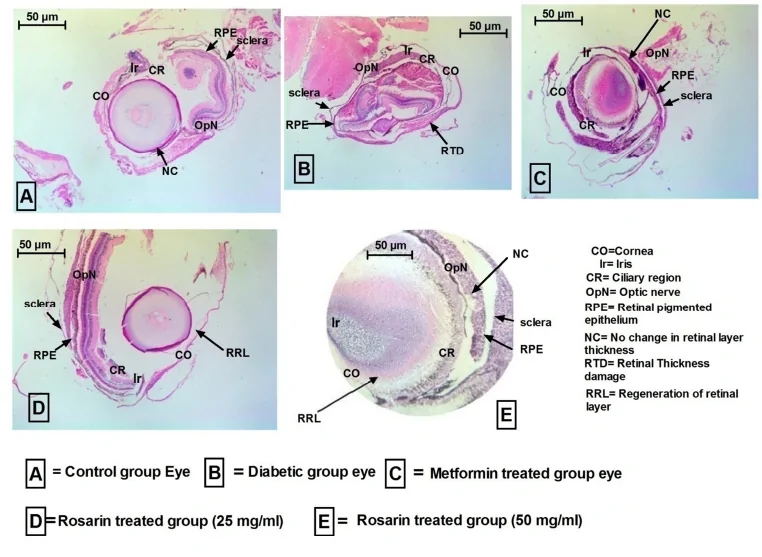
Histopathological photographs of Zebrafish retina layer in the negative control, diabetic control, rosarin and metformin-treated groups.

**Fig. (5):**
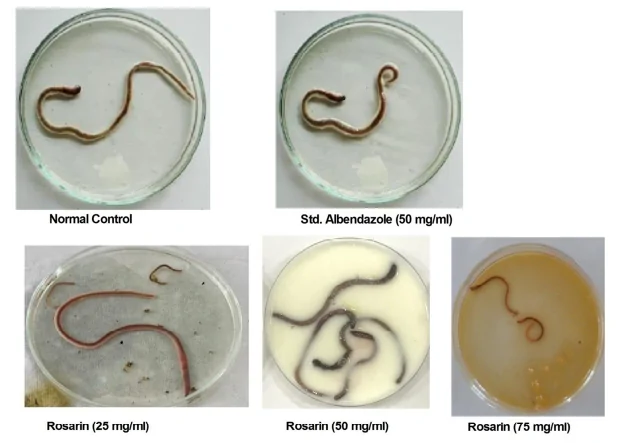
Effect of Rosarin on the length of *Pheretima posthuma* worms in centimeters.

**Table 1:** Influence of rosarin on body weight of zebrafish in anti-diabetic study.

**S. No**	**Name of the Group**	**Body Weight (g)**
1	Control	0.86 ± 0.03
2	Diabetic group	13.4 ± 0.11
3	Standard Metformin (50 mg/ml)	1.75 ± 0.05***
4	Rosarin low dose (25 mg/ml)	2.21 ± 0.04
5	Rosarin high dose (50 mg/ml)	2.0 ± 0.047***

**Note: ** Results are presented as Mean ± SEM, compared to the diabetes control group, and then tested using the Dunnett's test after a one-way ANOVA. The figures are statistically significant at ***p<0.001. 95% CI (1.953- 2.047) limits.

**Table 2:** Influence of rosarin on blood glucose level in zebrafish in anti-diabetic study.

**S. No**	**Name of the Group**	**Blood Glucose Level (mg/dl)**
1	Control	86.1 ± 1.85
2	Diabetic group	135.0 ± 3.14
3	Standard metformin (50 mg/ml)	79.2 ± 1.61***
4	Rosarin low dose (25 mg/ml)	90.9 ± 1.02
5	Rosarin high dose (50 mg/ml)	82.1 ± 0.59***

**Note: ** Results are presented as Mean ± SEM, compared to the diabetes control group, and then tested using the Dunnett's test after a one-way ANOVA. The figures are statistically significant at ***p<0.001. 95% CI (81.628- 82.572) limits.

**Table 3:** Effect of Rosarin on Liver enzymes (AST, ALT, ALP) of zebrafish.

**S. No**	**Name of the Group**	**AST** **IU/L**	**ALT** **IU/L**	**ALP** **IU/L**
1	Control	42.62 ± 1.31	46.51 ± 1.79	97.52 ± 1.37
2	Diabetic group	215.17 ± 1.75	148.23 ± 1.42	193.41 ± 1.56
3	Standard metformin (50 mg/ml)	142.32 ± 1.64***	92.46 ± 1.72***	127.52 ± 1.12***
4	Rosarin low dose (25 mg/ml)	175.48 ± 1.38*	125.72 ± 1.63	165.62 ± 1.35*
5	Rosarin high dose (50 mg/ml)	157.12 ± 1.92***	112.69 ± 1.95**	153.18 ± 1.46**

**Note: ** Results are presented as Mean ± SEM, compared to the diabetes control group, and then tested using the Dunnett's test after a one-way ANOVA. The figures are statistically significant at ***p<0.001, **p<0.01 and *p<0.1. 95% CI (81.628- 82.572) limits.

**Table 4:** Effect of Rosarin on length of worm in anthelminitic activity.

**S. No**	**Name of the Group**	**Length of Worm (cm)**
1	Control	14 ± 0.00
2	Albendazole (50 mg/ml)	8.9 ± 0.27***
3	Rosarin low dose (25 mg/ml)	13.0 ± 0.28
4	Rosarin medium dose (50 mg/ml)	12.0 ± 0.29
5	Rosarin high dose (75 mg/ml)	9.5 ± 0.36***

**Note: ** Results are presented as Mean ± SEM, compared to the diabetes control group, and then tested using the Dunnett's test after a one-way ANOVA. The figures are statistically significant at ***p<0.001. 95% CI (81.628- 82.572) limits.

**Table 5:** Effect of Rosarin on time of paralysis (minutes) and time of death (minutes) of worms in anthelminitic activity.

**S.No**	**Name of the Group**	**Time of Paralysis (Minutes)**	**Time of Death (Minutes)**
1	Control	51 ± 0..82	75 ± 1.64
2	Albendazole (50 mg/ml)	23 ± 0.60	42 ± 1.39
3	Rosarin low dose (25 mg/ml)	43 ± 0.57	61 ± 0.88
4	Rosarin medium dose (50 mg/ml)	33 ± 0.66	54 ± 1.2
5	Rosarin high dose (75 mg/ml)	22 ± 0.7.6***	40 ± 0.76***

**Note: ** Results are presented as Mean ± SEM, compared to the diabetes control group, and then tested using the Dunnett's test after a one-way ANOVA. The figures are statistically significant at ***p<0.001. 95% CI (81.628- 82.572) limits.

## Data Availability

The data and supportive information are available within the article.

## LIST OF ABBREVIATIONS

ml: Milliliter
mg/L: Milligram Per Liter
mg/ml: Milligram Per Milliliter
S.E.M: Standard Error of the Mean
p: Probability
°C: Degree Celsius
mM: Millimolar
g: Gram
mg/dl: Milligram per Deciliter
IU/L: International Units Per Litre
